# Extracellular Vesicles in Obesity: From Pathophysiological Mediators to Therapeutic Tools

**DOI:** 10.3390/ijms27073137

**Published:** 2026-03-30

**Authors:** Nikola Pavlović, Petar Todorović, Mirko Maglica, Andrea Kopilaš, Roko Šantić, Marko Kumrić, Marino Lukenda, Joško Božić

**Affiliations:** 1 Department of Pathophysiology, University of Split School of Medicine, 21000 Split, Croatia; nikola.pavlovic@mefst.hr (N.P.); roko.santic@mefst.hr (R.Š.); marko.kumric@mefst.hr (M.K.); 2 Laboratory for Cardiometabolic Research, University of Split School of Medicine, 21000 Split, Croatia; 3 Department of Anatomy, Histology and Embryology, University of Split School of Medicine, 21000 Split, Croatia; petar.todorovic@mefst.hr; 4 Department of Anatomy, School of Medicine, University of Mostar, 88000 Mostar, Bosnia and Herzegovina; mirko.maglica@mef.sum.ba; 5 Department of Otorhinolaryngology, University Hospital of Split, 21000 Split, Croatia; 6 Department of Family Medicine, Health Center Mostar, 88000 Mostar, Bosnia and Herzegovina; andrea.kopilas@mef.sum.ba; 7 Department of Cardiovascular Diseases, University Hospital of Split, 21000 Split, Croatia; 8 Ministry of Defence of the Republic of Croatia, 10000 Zagreb, Croatia; marino.lukenda@morh.hr

**Keywords:** extracellular vesicles, exosomes, obesity, metabolic syndrome, adipose tissue

## Abstract

Obesity is increasingly recognized as a disease of dysregulated intercellular communication rather than merely an energy imbalance. Extracellular vesicles (EVs), membrane-bound nanoparticles (30–1000 nm) released by nearly all cell types, act as central mediators of this pathological crosstalk. In obesity, hypertrophic adipocytes, pro-inflammatory macrophages, and dysfunctional endothelial cells secrete EVs carrying altered cargo, including pro-inflammatory miRNAs (e.g., miR-34a, miR-155), bioactive lipids, and stress proteins, which propagate systemic metabolic dysfunction. Adipose tissue-derived EVs impair hepatic fatty acid oxidation, promote steatohepatitis, suppress pancreatic beta-cell insulin secretion, induce skeletal muscle insulin resistance via PPARγ repression, and contribute to endothelial dysfunction and atherosclerosis. EV-mediated adipocyte–macrophage crosstalk reinforces chronic adipose inflammation. Circulating EVs also provide biomarkers: subpopulation ratios, miRNA signatures, and tissue factor-positive EVs reflect disease severity, predict cardiovascular risk, and monitor therapeutic responses, with machine learning enhancing diagnostic precision. Therapeutically, EVs from mesenchymal stem cells, Wharton’s jelly MSCs, adipose progenitors, and M2 macrophages reverse insulin resistance, hepatic steatosis, and adipose inflammation in preclinical models. Engineering strategies improve EV potency and tissue targeting, and Phase I trials confirm safety, though manufacturing and cost remain barriers. Preclinical and early clinical studies of MSC-EVs confirm a favorable safety profile, though manufacturing scalability and cost remain barriers to widespread clinical adoption. Overall, EVs represent both diagnostic tools and therapeutic vehicles in precision obesity medicine, offering a pathway from symptom management toward true disease remission.

## 1. Introduction

While obesity is traditionally viewed as an imbalance between energy intake and expenditure, emerging evidence reveals a more nuanced pathophysiology: obesity is fundamentally a disease of dysregulated intercellular communication. The global prevalence of obesity has tripled since 1975, and the condition now represents one of the leading preventable contributors to morbidity and mortality worldwide [1]. Conventional research paradigms have long centered on energy balance, adipocyte hypertrophy, and genetic susceptibility as the primary drivers of obesity-associated metabolic dysfunction [1,2]. Although these factors are undoubtedly important, they fail to fully account for the systemic, multi-organ nature of obesity complications, which include insulin resistance, non-alcoholic fatty liver disease, cardiovascular dysfunction, and chronic low-grade inflammation [3,4]. A critical gap persists in our understanding of how pathological signals emanating from dysfunctional adipose tissue are transmitted to anatomically distant organs to propagate metabolic disease. Extracellular vesicles (EVs) have recently emerged as compelling candidates to fill this gap, functioning as nanoscale intercellular messengers that coordinate metabolic crosstalk across tissues.

EVs are a heterogeneous family of membrane-bound particles, approximately 30–1000 nm in diameter, released by virtually all cell types into the extracellular space [5,6]. According to the updated nomenclature guidelines established by the International Society for Extracellular Vesicles (MISEV2023), EVs encompass small EVs (previously termed exosomes, ~30–150 nm), medium EVs (microvesicles, ~100–1000 nm), and large EVs, including apoptotic bodies [6]. These vesicles are generated through distinct biogenesis pathways: small EVs originate from the endosomal pathway via multivesicular bodies, whereas medium and large EVs bud directly from the plasma membrane [7]. Regardless of their subcellular origin, EVs encapsulate a molecularly diverse cargo that includes microRNAs (miRNAs), messenger RNAs, proteins, lipids, and metabolites, which are selectively packaged and can be functionally transferred to recipient cells [8]. Importantly, the lipid bilayer membrane of EVs protects their cargo from enzymatic degradation in the circulation, enabling long-range signaling between tissues.

In the context of obesity, EVs effectively function as a molecular “postal service,” ferrying disease-promoting cargo from expanded and inflamed adipose tissue to metabolically active organs, including the liver, pancreas, skeletal muscle, and vasculature. Adipocytes release EVs containing bioactive lipids that modulate macrophage activation and systemic inflammation [9]. Adipose tissue-derived EVs carry miRNAs such as miR-34a, which suppresses M2 macrophage polarization and amplifies adipose inflammation [10]. Conversely, adipose tissue macrophages (ATMs) in obese individuals secrete exosomes enriched in miRNAs, including miR-155, that impair insulin signaling in hepatocytes and myocytes, establishing a paracrine-to-endocrine axis of metabolic dysfunction [11]. Circulating exosomal miRNAs from obese subjects have been shown to modulate glucose and lipid metabolism in distant target cells, underscoring the systemic reach of EV-mediated communication [12,13]. Moreover, seminal work by Thomou et al. demonstrated that adipose tissue is a major source of circulating exosomal miRNAs and that these miRNAs regulate gene expression in other tissues, including the liver, thereby establishing adipose-derived EVs as bona fide endocrine mediators [14].

Crucially, the role of EVs in obesity extends beyond that of passive biomarkers. Functional transfer experiments have provided direct evidence that EVs act as strong mechanistic candidates for causal involvement in metabolic disease propagation as supported by preclinical gain- and loss-of-function studies. Injection of ATM-derived exosomes from obese mice into lean recipients induces glucose intolerance and insulin resistance, phenocopying key features of metabolic syndrome [11]. Similarly, adipose-derived exosomal miRNAs transferred to lean animals alter hepatic glucose output and lipid metabolism [12]. Adipocyte-derived EVs also regulate pancreatic β-cell survival and function, providing a direct molecular link between adipose dysfunction and impaired insulin secretion. Recent studies have further revealed that energetically stressed adipocytes transport mitochondrial components via EVs to cardiomyocytes, potentially contributing to obesity-associated cardiomyopathy [15]. Exosomal miR-29a from obese ATMs has been independently shown to drive insulin resistance in adipocytes and hepatocytes through suppression of insulin receptor substrate signaling [16]. These findings collectively demonstrate that EVs are not merely correlates of obesity but active participants in its pathogenesis.

**Figure 1:**
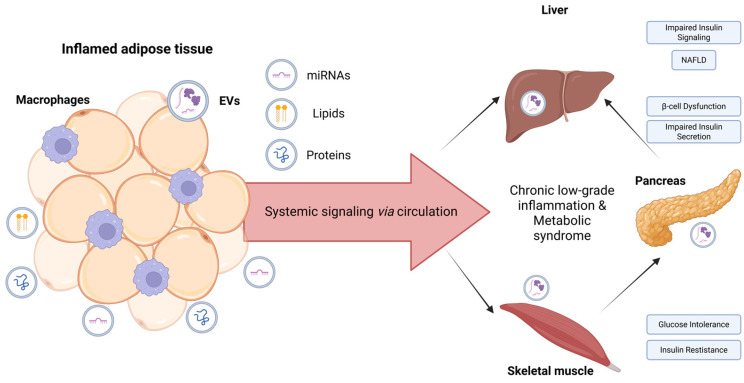
Role of extracellular vesicles (EVs) from inflamed adipose tissue in systemic metabolic dysfunction. Hypertrophic adipocytes and pro-inflammatory macrophages in inflamed adipose tissue release EVs carrying molecular cargo, including miRNAs, lipids, and proteins. These EVs enter the circulation and mediate systemic signaling, contributing to chronic low-grade inflammation and metabolic syndrome. Tissue-specific uptake of adipose-derived EVs affects multiple organs: in the liver, they impair insulin signaling and promote non-alcoholic fatty liver disease (NAFLD); in the pancreas, they induce β-cell dysfunction and reduce insulin secretion; and in skeletal muscle, they cause insulin resistance and glucose intolerance. This schematic represents a conceptual model based on current preclinical and emerging clinical evidence; arrows denote proposed mechanistic relationships rather than fully established causal pathways. Created in BioRender. Kumrić, M. (2026) https://BioRender.com/ptwxu2c (accessed on 2 February 2026).

The recognition of EVs as causative mediators of obesity-related metabolic dysfunction opens a compelling translational avenue: if pathological EVs drive disease, then engineered or therapeutically modified EVs may be harnessed to reverse it. Indeed, EVs from adipose-derived stem cells have already demonstrated the capacity to attenuate adipose inflammation and promote browning of white adipose tissue in preclinical models [17]. Furthermore, the ability to load EVs with specific miRNAs or proteins positions them as highly versatile drug delivery vehicles with inherent biocompatibility and tissue-targeting properties [18]. Meanwhile, circulating exosomal miRNA signatures are being explored as minimally invasive biomarkers for obesity stratification and metabolic risk assessment [19]. This review synthesizes the current understanding of EV biology in the pathophysiology of obesity, examines the molecular cargo responsible for inter-organ metabolic crosstalk, and evaluates the emerging potential of engineered EVs as a precision therapeutic modality for obesity-related metabolic disease. This review is organized around three complementary objectives: (1) to synthesize the mechanistic evidence linking EV-mediated intercellular communication to the pathophysiology of obesity and its multi-organ complications; (2) to evaluate the diagnostic and prognostic potential of circulating EVs as biomarkers of metabolic disease severity and therapeutic response; and (3) to assess the translational landscape of engineered EV therapeutics, from preclinical proof-of-concept to early clinical investigation. As illustrated in Figure 1, EVs released from inflamed adipose tissue mediate systemic signaling that contributes to chronic inflammation and metabolic dysfunction in the liver, pancreas, and skeletal muscle.

## 2. EV Production, Cargo Composition, and Their Alterations in Obesity

EVs represent a heterogeneous population of membrane-enclosed particles that are classified by size, biogenesis, and molecular composition. Current consensus guidelines (MISEV2023) categorize EVs into three principal subtypes [6]. Small EVs, historically referred to as exosomes, range from approximately 30 to 150 nm in diameter and originate from the endosomal compartment; inward budding of the limiting membrane of late endosomes generates intraluminal vesicles within multivesicular bodies (MVBs), which are subsequently released into the extracellular space upon MVB fusion with the plasma membrane [7]. Medium EVs, or microvesicles, span 100–1000 nm and are generated by direct outward budding and fission of the plasma membrane, a process that involves cytoskeletal reorganization and asymmetric redistribution of membrane phospholipids [8]. Apoptotic bodies, the largest EV subclass (>1000 nm), arise from membrane blebbing during programmed cell death and carry intact organelles and nuclear fragments [7]. Although each subtype possesses distinct biogenetic hallmarks, considerable overlap in size and surface markers persists, necessitating careful operational definitions in experimental studies [6].

Obesity profoundly alters EV dynamics at multiple levels. Circulating concentrations of adipocyte-derived EVs are significantly elevated in obese compared to lean individuals and correlate with markers of metabolic dysfunction, including insulin resistance and hepatic steatosis [20]. Importantly, this increase in EV output is not merely a consequence of expanded adipose tissue mass. In vitro studies demonstrate that hypertrophic adipocytes release greater numbers of EVs on a per-cell basis compared to smaller adipocytes, indicating that cellular stress intrinsic to adipocyte enlargement drives enhanced vesicle biogenesis [21]. These observations are clinically reinforced by the finding that circulating exosomal miRNA profiles and EV-associated lipid signatures normalize following bariatric surgery-induced weight loss, paralleling improvements in insulin sensitivity [22,23]. Thus, EV overproduction in obesity reflects a pathologically activated secretory program rather than a passive scaling phenomenon.

Beyond increased quantity, the molecular cargo of EVs undergoes qualitative reprogramming in obesity. The miRNA content of obese adipose-derived EVs is particularly well characterized and shows a consistent shift toward pro-inflammatory and metabolism-disrupting species. Exosomal miR-34a, secreted by hypertrophic adipocytes, inhibits M2 macrophage polarization in recipient tissue-resident macrophages, thereby amplifying the pro-inflammatory milieu within adipose depots [10]. Adipose tissue macrophages in the obese state secrete exosomes enriched in miR-155, which directly suppresses peroxisome proliferator-activated receptor gamma (PPARγ) expression and impairs insulin signaling in hepatocytes and myocytes [11]. Circulating exosomal miR-122 and miR-192, both significantly upregulated in obese subjects, modulate hepatic lipogenesis and gluconeogenesis in distant target organs [12]. Additionally, adipocyte-derived exosomal miR-27a represses PPARγ in skeletal muscle, establishing a direct molecular link between adipose-derived vesicles and peripheral insulin resistance [24]. The downstream functional consequences of this altered cargo are far-reaching; for instance, adipocyte-derived EVs carrying this dysregulated miRNA repertoire have been shown to impair pancreatic β-cell survival and insulin secretory function [25].

The proteomic and lipidomic composition of EVs is similarly remodeled in obesity. Adipocyte-derived EVs from obese subjects show enrichment in stress-response proteins, including markers of endoplasmic reticulum stress and mitochondrial dysfunction, whereas proteins involved in healthy lipid metabolism and insulin signaling are diminished [15]. Notably, energetically stressed adipocytes package mitochondrial components into EVs, suggesting that vesicular export of damaged organellar material serves as a cellular stress-relief mechanism with potential downstream consequences for recipient tissues [15]. The lipid composition of obese-derived EVs is also significantly altered. Adipocytes release EVs enriched in ceramides and bioactive lipid species that activate pro-inflammatory pathways in macrophages [9]. EV-associated lipid cargo in obese individuals further includes alterations in cholesterol efflux capacity, suggesting that obesity disrupts the normal lipid-handling function of circulating vesicles [26]. In addition, obesity is associated with elevated production of procoagulant microparticles that promote endothelial dysfunction and may contribute to the prothrombotic state characteristic of this condition [27]. This lipid and surface remodeling of the EV membrane not only alters its signaling potential but also influences uptake efficiency and tropism in target tissues [28]. The key EV cargo alterations in obesity and their functional effects are summarized in Table 1. Together, these cargo alterations reflect a shift from homeostatic to pathological EV signaling in obesity, with miRNAs representing the most extensively characterized mediators of inter-organ metabolic dysfunction.

The pathogenicity of obesity-associated EVs is further modulated by tissue of origin and biological sex. EVs derived from visceral adipose tissue (VAT) exhibit a markedly more pro-inflammatory cargo profile compared to those originating from subcutaneous adipose tissue (SAT), consistent with the established greater metabolic risk conferred by visceral adiposity [28]. VAT-derived EVs carry higher concentrations of inflammatory mediators that promote endothelial dysfunction and vascular inflammation [28]. Sex-based differences in EV biology are also emerging as an important modifier of obesity-related metabolic risk. Normal-weight and overweight women exhibit distinct plasma EV concentrations and miRNA cargo profiles, suggesting that biological sex influences both EV release and content [29]. Pre-menopausal women appear to have a more favorable circulating EV profile compared to postmenopausal women, an observation potentially linked to estrogen-mediated regulation of miRNA expression in the cardiovascular system [30]. From a clinical standpoint, the distinctive circulating EV miRNA signatures associated with obesity present an opportunity for early disease detection. Plasma exosomal miRNA profiles can discriminate obese from lean individuals and correlate with metabolic parameters before conventional biomarkers such as fasting glucose or hemoglobin A1c become abnormal, positioning EV-based liquid biopsies as promising tools for presymptomatic metabolic risk stratification [19].

## 3. EV-Mediated Adipocyte-Macrophage Crosstalk as Driver of Adipose Tissue Inflammation

Chronic low-grade adipose tissue inflammation is a defining feature of obesity and a critical link between excess adiposity and systemic metabolic dysfunction. While early paradigms attributed adipose inflammation primarily to increased secretion of soluble cytokines such as TNF-α and IL-6 [3], this framework incompletely explains the stability, amplification, and cell-type specificity of inflammatory remodeling. Accumulating evidence now identifies extracellular vesicles (EVs) as central orchestrators of adipocyte–macrophage communication, positioning EV-mediated signaling as a primary mechanism driving immune reprogramming in obese adipose tissue [11].

### 3.1. EV Signaling in Lean Adipose Tissue: Maintenance of Immune Homeostasis

In lean adipose tissue, EV secretion occurs at relatively low levels and is characterized by an anti-inflammatory and metabolically supportive cargo profile. Adipocyte-derived EVs under physiological conditions contain miRNAs and proteins that promote insulin sensitivity, mitochondrial function, and immune tolerance [14]. These vesicles contribute to the maintenance of an adipose-resident macrophage population dominated by alternatively activated (M2-like) macrophages, which secrete IL-10, support angiogenesis, and facilitate extracellular matrix remodeling necessary for healthy tissue expansion. EV uptake by macrophages in this context does not activate canonical inflammatory pathways such as NF-κB or STAT signaling, allowing adipose tissue to dynamically adapt to nutritional fluctuations without triggering immune activation. Thus, in the lean state, EV-mediated adipocyte–macrophage communication serves a homeostatic role, reinforcing immune quiescence and preserving adipose tissue metabolic flexibility [11,14,31].

### 3.2. Obesity-Induced Reprogramming of Adipocyte EVs and Macrophage Polarization

Obesity profoundly alters both the quantity and molecular composition of adipocyte-derived EVs. Hypertrophic adipocytes experience mechanical stress, lipotoxicity, oxidative stress, and endoplasmic reticulum (ER) stress, all of which stimulate EV biogenesis and release. These obesity-associated EVs are enriched in pro-inflammatory miRNAs that actively reprogram recipient macrophages toward a classically activated (M1-like) phenotype [32,33]. Among the best-characterized mediators is miR-155, which is markedly enriched in EVs released from obese adipocytes. Upon transfer to macrophages, miR-155 directly targets SHIP1, a phosphatase that functions as a critical negative regulator of STAT signaling. Loss of SHIP1 removes an essential inhibitory constraint on STAT3 activation, resulting in enhanced transcription of pro-inflammatory genes, including IL1B and IL6. Functionally, macrophages exposed to miR-155-rich adipocyte EVs exhibit increased cytokine secretion, nitric oxide production, and impaired insulin-sensitizing capacity, hallmarks of an M1 inflammatory phenotype [33,34,35]. In parallel, obese adipocyte-derived EVs suppress anti-inflammatory macrophage differentiation. EV-associated miR-34a inhibits macrophage polarization toward the M2 state by targeting MSI2, thereby disrupting Wnt/β-catenin signaling required for alternative activation. Notably, this suppression persists even in the presence of canonical M2-polarizing cytokines such as IL-10 and IL-13, indicating that EV cargo exerts dominant control over macrophage fate decisions. This mechanism provides a molecular explanation for the failure of anti-inflammatory cytokine environments to restore immune balance in obese adipose tissue [10,35,36].

### 3.3. EVs as Sufficient and Necessary Drivers of Adipose Inflammation

A defining strength of the EV paradigm is the availability of direct causal evidence. Multiple proof-of-concept studies demonstrate that EVs are not merely markers or amplifiers of inflammation, but sufficient and necessary mediators of metabolic dysfunction. Administration of adipose tissue-derived EVs from obese donors into lean mice induces macrophage infiltration, inflammatory gene expression, and systemic glucose intolerance [37]. Conversely, delivery of EVs isolated from lean adipose tissue into obese mice attenuates adipose inflammation and improves insulin sensitivity without altering body weight or caloric intake [3,22,34,38]. These gain- and loss-of-function experiments establish EVs as upstream regulators of adipose immune remodeling, independent of adiposity per se. Importantly, they demonstrate that EV-mediated transfer of genetic information is sufficient to impose a pathological macrophage phenotype, consistent with a causal, rather than merely reactive, role for EVs in adipose inflammation.

### 3.4. Self-Reinforcing EV-Driven Feedback Loops in Obese Adipose Tissue

Once initiated, EV-mediated adipocyte–macrophage crosstalk is sustained through multiple interconnected feedback loops that stabilize chronic inflammation.

In the first loop, pro-inflammatory EVs released by hypertrophic adipocytes induce M1 macrophage polarization, leading to increased secretion of TNF-α and IL-1β. These cytokines exacerbate adipocyte ER stress and insulin resistance, further stimulating EV release and reinforcing inflammatory cargo loading [39].

A second loop is driven by adipose tissue hypoxia. As adipocyte hypertrophy outpaces angiogenic capacity, hypoxic signaling activates HIF-1α, which promotes the release of EVs enriched in pro-inflammatory and pro-fibrotic miRNAs. These EVs amplify macrophage activation and extracellular matrix deposition, contributing to adipose tissue stiffening and further metabolic dysfunction [40,41].

A third loop involves macrophage-derived EVs. Inflammatory macrophages secrete EVs that impair insulin signaling in adipocytes, disrupt lipid storage, and promote lipolysis. This metabolic stress feeds back to adipocytes, increasing EV secretion and perpetuating the inflammatory circuit [9].

Collectively, these feedback loops establish an EV-driven network that locks adipose tissue into a state of chronic inflammation, even in the absence of acute inflammatory triggers.

### 3.5. Paradigm Shift and Therapeutic Implications

This EV model fundamentally reframes obesity associated inflammation. Rather than viewing macrophage activation as a downstream response to elevated cytokines or free fatty acids, EVs emerge as the primary vectors that establish, propagate, and stabilize pathological immune phenotypes. This framework explains why therapies targeting individual cytokines have yielded limited metabolic benefit: without disrupting EV-mediated genetic reprogramming, macrophage identity remains fixed in an inflammatory state. By identifying EV biogenesis, cargo selection, and cellular uptake as upstream regulatory nodes, this model opens new therapeutic avenues. Strategies aimed at limiting pathogenic EV release, modifying EV cargo, or blocking EV uptake by macrophages may allow restoration of adipose immune homeostasis and interruption of obesity driven metabolic disease progression [42].

## 4. EVs and Organ-Specific Metabolic Complications

Obesity is characterized by the simultaneous development of metabolic dysfunction across multiple organs, including the liver, pancreas, skeletal muscle, and vasculature. This multi-organ involvement is often treated as a collection of parallel pathologies [43]; however, emerging evidence suggests that EVs derived from dysfunctional adipose tissue act as coordinated endocrine-like signals that propagate metabolic derangements systemically. Through organ-specific uptake, adipose-derived EVs translate adipose inflammation into widespread metabolic disease [11]. An overview of the principal EV subpopulations, their organ-specific targets, and the resulting pathological consequences is presented in Table 2.

### 4.1. Liver: EV-Mediated Progression Toward Steatosis and Fibrosis

The liver is a primary target of adipose-derived EVs. In obesity, EVs released from visceral adipose tissue are enriched in miRNAs and lipids that impair hepatic fatty acid oxidation and promote lipid accumulation. Experimental studies demonstrate that obese adipocyte-derived EVs suppress hepatic AMPK signaling and downregulate genes involved in mitochondrial β-oxidation, leading to triglyceride accumulation and hepatic steatosis. Sustained EV exposure promotes inflammatory and fibrotic gene expression in hepatic stellate cells, positioning EVs as upstream mediators in the transition from simple steatosis to metabolic dysfunction-associated steatohepatitis (MASH) [44,45].

### 4.2. Pancreas: β-Cell Dysfunction and Impaired Insulin Secretion

Pancreatic β-cells are highly sensitive to obesity-associated EV signaling. Adipocyte-derived EVs from obese donors inhibit glucose-stimulated insulin secretion and induce β-cell stress responses. EV-associated miRNAs impair insulin granule exocytosis, reduce mitochondrial ATP generation, and promote β-cell dedifferentiation. In vivo models show that chronic exposure to obese adipose EVs exacerbates β-cell dysfunction independently of hyperglycemia, suggesting that EVs act as early mediators of pancreatic failure in obesity [46,47,48].

### 4.3. Skeletal Muscle: Insulin Resistance and Metabolic Inflexibility

Skeletal muscle, the major site of insulin-stimulated glucose disposal, is another critical EV target. Obese adipose-derived EVs impair glucose uptake in myocytes by suppressing insulin receptor signaling and attenuating AMPK activation. This results in reduced glucose transport, diminished fatty acid oxidation, and loss of metabolic flexibility. EV mediated delivery of inflammatory miRNAs contributes to mitochondrial dysfunction and reduced exercise responsiveness, linking adipose inflammation to systemic insulin resistance [49,50,51].

### 4.4. Vasculature: Endothelial Dysfunction and Atherosclerosis

Adipose-derived EVs also exert potent effects on the vascular endothelium. Obesity associated EVs suppress endothelial nitric oxide synthase (eNOS) activity, increase oxidative stress, and promote endothelial inflammation. In macrophages within the vascular wall, uptake of visceral adipose tissue-derived EVs downregulates cholesterol efflux transporters ABCA1 and ABCG1, impairing reverse cholesterol transport [52]. This promotes foam cell formation, lipid accumulation, and progression of atherosclerotic lesions, directly linking adipose EV signaling to cardiovascular disease [28,32].

### 4.5. Autocrine and Paracrine EV Signaling Within Adipose Tissue

Beyond endocrine effects, EVs also mediate autocrine and paracrine communication within adipose tissue. Hypertrophic adipocytes exchange EVs with neighboring adipocytes, synchronizing metabolic dysfunction, insulin resistance, and inflammatory signaling across the depot. This coordinated intra-adipose communication accelerates tissue dysfunction and amplifies EV release into the circulation [53].

### 4.6. Clinical Implications: Coordinated, Not Random, Multi-Organ Disease

These findings collectively support a paradigm shift in our understanding of obesity-related metabolic disease. Rather than viewing complications such as hepatic steatosis, insulin resistance, β-cell dysfunction, and vascular pathology as independent, parallel events, emerging evidence positions adipose-derived EVs as central orchestrators of a systemic network of dysfunction. By acting as endocrine-like messengers, EVs propagate signals of metabolic stress from hypertrophic adipose tissue to distant organs, while simultaneously coordinating intra-adipose communication through autocrine and paracrine signaling [54].

This networked view of metabolic disease has several important clinical implications. First, it emphasizes that interventions targeting a single organ or pathway may be insufficient; therapeutic strategies must consider the multi-organ integration of metabolic stress mediated by EVs [48].

Second, circulating EVs could serve as biomarkers of systemic metabolic burden, reflecting not only adipose tissue inflammation but also early dysfunction in the liver, pancreas, skeletal muscle, and vasculature [55].

Third, modulating EV biogenesis, release, or cargo represents a promising avenue to interrupt the propagation of metabolic derangements, potentially preventing or mitigating multiple downstream complications simultaneously [56].

Ultimately, framing obesity as a coordinated EV-driven network of disease underscores the interconnectedness of metabolic organs and highlights the importance of systemic, rather than organ-isolated, approaches in both research and clinical management. Recognizing this interdependence may lead to more effective strategies to prevent the cascade of metabolic complications that characterizes obesity and to the development of therapies that restore communication balance across the entire metabolic network [56,57,58]. The cellular origins, pathological roles, and therapeutic opportunities associated with each EV subpopulation are detailed in Table 2.

## 5. EV Subpopulations and Cell-Type-Specific Roles in Obesity

The heterogeneity of EVs extends beyond differences in size and biogenesis; distinct cellular origins endow EV subpopulations with specialized molecular cargo and correspondingly distinct pathophysiological roles in obesity [6]. Adipocyte-derived EVs (AEVs) constitute the most extensively studied subpopulation in the context of metabolic disease. Hypertrophic adipocytes release EVs carrying pro-inflammatory miRNAs, most notably miR-34a, which suppresses anti-inflammatory M2 macrophage polarization and perpetuates adipose tissue inflammation [10]. AEVs from visceral adipose tissue (VAT) display a markedly more inflammatory cargo profile than those from subcutaneous adipose tissue (SAT), carrying higher concentrations of pro-inflammatory mediators that promote endothelial activation and vascular dysfunction [28]. Beyond inflammatory signaling, AEVs serve as vehicles for interorgan transfer of metabolic stress: energetically stressed adipocytes export damaged mitochondrial components to cardiomyocytes via EVs, potentially contributing to obesity-associated cardiac dysfunction. AEVs also impair pancreatic β-cell survival and insulin secretory capacity, establishing a direct vesicular link between adipose dysfunction and the progression toward type 2 diabetes [25].

Macrophage-derived EVs represent a second critical subpopulation whose functional polarity shifts dramatically in obesity. Adipose tissue macrophages (ATMs) in the obese state adopt a predominantly pro-inflammatory (M1-like) phenotype and secrete exosomes enriched in miR-155 and miR-29a, which impair insulin receptor signaling in hepatocytes, adipocytes, and skeletal muscle cells [11,16]. Crucially, this effect is phenotype-dependent: exosomes from M1-polarized ATMs transfer insulin resistance to lean recipients, whereas EVs from anti-inflammatory M2 macrophages exert opposing, insulin-sensitizing effects [11]. This functional dichotomy has been therapeutically exploited in preclinical models. Exosomes derived from adipose-derived stem cells promote M2 macrophage polarization and stimulate browning of white adipose tissue in obese mice, resulting in improved metabolic parameters [17]. Furthermore, M2 macrophage-derived exosomes carrying miR-690 have been shown to directly improve insulin sensitivity in obese mice by suppressing Nadk-mediated metabolic pathways, demonstrating that macrophage-derived EVs can be harnessed as anti-obesity therapeutic vehicles [59].

Platelet-derived microvesicles (PMVs) are significantly elevated in obese individuals and constitute a major link between obesity and cardiovascular thrombotic risk. PMVs from obese subjects are characterized by altered proteomic profiles and enhanced capacity to activate endothelial cells, promoting a pro-adhesive and pro-inflammatory vascular phenotype [27]. Beyond their direct effects on endothelium, PMVs participate in broader vascular inflammatory circuits by transferring bioactive mediators that can either amplify tissue injury or, under certain conditions, promote resolution [60]. A particularly important subset of circulating EVs in obesity comprises those bearing tissue factor (TF), the principal initiator of the extrinsic coagulation cascade. TF-positive EVs, derived from activated platelets, monocytes, and endothelial cells, are enriched in the circulation of obese individuals and confer enhanced procoagulant potential [61]. These TF-bearing vesicles are thought to represent a key mechanistic link between obesity and the elevated incidence of venous thromboembolism and arterial thrombotic events observed in this population [62].

Endothelial microparticles (EMPs), shed from activated or damaged endothelial cells, serve as both markers and mediators of vascular dysfunction in obesity. EMPs carry endothelin-1 and other vasoactive molecules that directly injure recipient endothelial cells, amplifying vascular dysfunction in a paracrine feed-forward manner [63]. Phenotyping studies of circulating EVs in obese individuals have revealed that large EVs of endothelial and monocytic origin are enriched in macrophage migration inhibitory factor (MIF), a potent pro-inflammatory cytokine that further exacerbates metabolic inflammation [64]. The convergence of elevated PMVs, TF-positive EVs, and EMPs in obese individuals creates a reinforcing pro-thrombotic and pro-inflammatory vascular milieu that substantially exceeds the pathogenic contribution of any single subpopulation [28,65].

Apoptotic bodies released from dying adipocytes within expanding adipose depots represent an additional, often underappreciated, EV subpopulation. Adipocyte death in obesity triggers a robust pro-inflammatory response, including the formation of crown-like structures in which recruited macrophages surround and engulf remnants of dead adipocytes, undergoing metabolic activation in the process [66]. The vesicular debris released during this process carries bioactive lipids and damage-associated molecular patterns (DAMPs) that activate pattern recognition receptors on macrophages, perpetuating a cycle of immune activation and further adipocyte loss [9,62]. Intriguingly, recent work has demonstrated that apoptotic vesicles also possess regenerative potential; in a murine model of type 2 diabetes, transplanted apoptotic vesicles restored liver macrophage homeostasis and improved glucose metabolism, suggesting context-dependent immunomodulatory properties of this EV subclass [65].

The delineation of EV subpopulations with distinct pathogenic roles carries significant therapeutic implications. If specific subpopulations are identified as principal drivers of defined complications, for instance, TF-positive EVs in thrombotic risk or M1 macrophage-derived exosomes in insulin resistance, then strategies can be developed to selectively deplete or neutralize these vesicles while sparing beneficial EV populations [18]. Conversely, the protective properties of certain subpopulations, such as M2 macrophage-derived exosomes or stem cell-derived EVs, can be leveraged to engineer therapeutic vesicles with tailored anti-inflammatory and insulin-sensitizing cargo [17,59]. Realizing this precision approach will require continued refinement of EV isolation and characterization methodologies to reliably distinguish subpopulations by cellular origin and functional potential [5,6]. Nevertheless, the emerging map of EV subpopulation-specific roles in obesity provides a rational foundation for the development of targeted vesicle-based diagnostics and therapeutics.

## 6. EVs as Biomarkers for Obesity Severity and Prognosis

The concept of the “liquid biopsy” has transformed diagnostic medicine, and circulating extracellular vesicles represent one of its most promising frontiers in metabolic disease. Unlike static serum analytes, EVs are dynamically released by metabolically stressed tissues, carry tissue-specific molecular cargo, and reflect the real-time functional state of their cells of origin. In the context of obesity, this translates into a uniquely informative biomarker platform: non-invasive, readily sampled from peripheral blood, and capable of simultaneously capturing information about adipose tissue, the liver, the vasculature, and immune compartments [32,67].

### 6.1. Total EV Count and Subpopulation Dynamics

Circulating EV concentrations positively correlate with BMI, visceral adiposity, and markers of metabolic risk. Meta-analytical data demonstrate that individuals with obesity exhibit significantly elevated total plasma EV levels compared with lean controls, with concentrations further increasing in those who progress to metabolic syndrome or type 2 diabetes mellitus (T2DM) [68]. Importantly, not all EV subpopulations tell the same story. The ratio of platelet-derived microvesicles (PMVs) to total EVs serves as an indicator of thrombotic risk: in obese patients, decreased levels of CD41+ platelet EVs alongside increased phosphatidylserine and Factor V expression signal a prothrombotic state that may contribute to elevated cardiovascular risk [69]. Endothelial microparticle (EMP) concentrations, identified by CD144+ or CD31+ surface markers, are elevated in adults with obesity and inversely correlate with endothelial fibrinolytic capacity, as evidenced by impaired tissue-type plasminogen activator (t-PA) release in response to bradykinin challenge [70]. This makes EMPs a candidate biomarker of endothelial dysfunction, preceding overt clinical cardiovascular disease. At the inflammatory nexus, the ratio of M1 macrophage-derived EVs to M2 macrophage-derived EVs within adipose tissue reflects the degree of adipose inflammation: obese adipose tissue macrophage EVs enriched in pro-inflammatory miR-155 and miR-27a drive insulin resistance and suppress healthy M2 polarization, while EVs from lean-state macrophages carry miR-690 and are inherently anti-inflammatory [24,71].

### 6.2. miRNA Cargo as a Precision Biomarker Layer

Among the molecular constituents of EVs, miRNA signatures have attracted the greatest interest as biomarkers due to their stability, tissue specificity, and functional relevance to metabolic pathways. Several miRNA species merit particular attention in obesity-related diagnostic contexts.

miR-122 and miR-192 are predominantly hepatocyte-derived and are enriched in circulating EVs in proportion to disease severity in metabolic dysfunction-associated steatotic liver disease (MASLD) and MASH. When hepatocyte-specific EVs are isolated using anti-asialoglycoprotein receptor 1 (ASGR1) immunoprecipitation, miR-122, miR-192, and miR-128-3p demonstrate significantly higher expression in MASH subjects compared with controls (*p* = 0.012, *p* = 0.013, and *p* = 0.032, respectively), outperforming global EV or total cell-free RNA analysis [72]. These EV-miRNA levels also correlate with liver fibrosis staging and hepatic inflammation scores, positioning them as non-invasive alternatives to liver biopsy for MASH staging and progression monitoring [73].

miR-27a and miR-27b are elevated in obese plasma EVs, particularly those derived from adipocytes and adipose tissue macrophages, and predict insulin resistance by repressing PPARγ signaling in skeletal muscle and hepatocytes [24]. Adipocyte-derived EV miR-27a has been shown to induce insulin resistance in skeletal muscle through this mechanism, and its plasma levels correlate with HOMA-IR scores in human cohorts [74]. miR-155 from obese adipose tissue macrophage EVs impairs both hepatocyte and pancreatic beta-cell insulin signaling, suppressing glucose-stimulated insulin secretion via the miR-155–MAFB axis, making it a candidate biomarker of adipose-macrophage activation and beta-cell functional compromise [47,71].

Visceral fat-specific EV miRNA signatures have also been identified through comprehensive profiling studies. miR-222, miR-23b, miR-4429, miR-148b, and miR-4269 are dysregulated in adipose tissue-derived EVs (AdEVs) and are associated with obesity-related comorbidities, including chronic inflammation and fibrosis [75]. EV-derived let-7a is significantly downregulated in obese subjects, with levels inversely correlating with BMI, and may carry prognostic relevance beyond adiposity itself [76]. A high-fat diet mouse model demonstrated concurrent increases in plasma EV miR-122, miR-192, miR-27a-3p, and miR-27b-3p, providing a multi-marker panel that recapitulates the human obesity phenotype at the molecular level [11].

### 6.3. Tissue Factor-Positive EVs and Cardiovascular Prognosis

Tissue factor (TF)-positive EVs, shed predominantly by endothelial cells, monocytes, and smooth muscle cells under inflammatory conditions, are elevated in obese patients and predict adverse cardiovascular outcomes. The procoagulant activity of TF+ EVs has been linked to thrombotic risk in obesity-associated cardiovascular disease, and circulating EV levels quantified by flow cytometry are strongly associated with cardiovascular risk markers, including blood pressure, atherogenic dyslipidemia, and endothelial dysfunction indices [77]. The PCSK9 pathway further modulates EV release from atherosclerosis-relevant cells (platelets, endothelium, neutrophils, and macrophages) in obese adults, suggesting a regulatory intersection between hypercholesterolemia and EV biology [69].

### 6.4. EVs as Predictors of Therapeutic Response

Beyond diagnosis, EV profiles hold considerable promise for predicting and monitoring treatment response. Circulating EV miRNA composition changes following weight loss interventions, making EVs potentially valuable as dynamic biomarkers of metabolic improvement. Pre-operative EV profiles may predict the magnitude of metabolic benefit following bariatric surgery: platelet- and endothelial-derived EV subpopulations, along with inflammatory biomarkers, are significantly altered by substantial weight loss and track the normalization of subclinical cardiac dysfunction in the post-bariatric period [78]. Similarly, GLP-1 receptor agonist therapy modulates adipose tissue EV secretion and cargo, potentially providing a pharmacodynamic readout of drug effect in adipose tissue that is not captured by traditional glycemic indices.

The EV-based MASLD staging framework has recently been augmented by machine learning (ML) approaches. EV mean size and plasma concentration, when combined with traditional clinical parameters and analyzed using explainable artificial intelligence models, demonstrated robust predictive performance for steatosis severity staging in MASLD patients, underscoring the potential of multi-parameter EV platforms for precision hepatology [73]. Similarly, an EV machine learning platform (EVMAP) combining microflow cytometry-derived EV fluorescence, concentration, and size data with clinical metadata has been shown to generate disease-specific prediction models with clinically meaningful area-under-the-curve performance [79].

### 6.5. Sex and Age as Modulators of the EV Biomarker Profile

Biological sex and age substantially modify the EV biomarker landscape in obesity. Pre-menopausal women carry a comparatively lower adipose-inflammatory EV burden than age-matched obese men, consistent with the cardioprotective effects of estrogens on adipose tissue macrophage polarization and vascular biology. Following menopause, this protection is attenuated, and circulating EV profiles shift toward patterns more similar to those observed in obese males. Age is an independent determinant of EV composition: adipose progenitor cells from middle-aged subjects secrete EVs that lack miR-145-5p, leading to impaired suppression of M1 macrophage polarization in adipose tissue via L-selectin/NF-κB signaling, a mechanism driving the heightened susceptibility to midlife obesity [80]. These age- and sex-related modifiers must be accounted for in biomarker interpretation and in the establishment of reference ranges.

### 6.6. Toward a Comprehensive EV Obesity Biomarker Panel

The convergent evidence positions EVs as the foundation of what might be envisioned as a comprehensive “obesity metabolic panel”, an integrated, non-invasive multi-marker approach that simultaneously interrogates hepatic (miR-122, miR-192), adipose-inflammatory (miR-155, miR-27a/b), vascular (EMP count, TF+ EVs), and prognostic (PMV/EMP ratios) dimensions of the obesity phenotype. Multiparametric EV frameworks incorporating bulk proteomics, single-vesicle interrogation, and miRNA profiling from matched adipose and plasma samples have demonstrated the co-enrichment of adiponectin, perilipin, CD63, and PPARγ in circulating adipose tissue-derived EVs, validating their tissue specificity and diagnostic informativeness [81]. The integration of ML algorithms with high-throughput EV phenotyping platforms will likely accelerate the translation of EV biomarker panels from research to clinical diagnostics, enabling real-time monitoring of disease trajectory and therapeutic response.

## 7. Engineering EVs as Therapeutic Agents—From Design to Clinical Translation

The pathophysiological evidence reviewed in preceding sections establishes that obesity fundamentally dysregulates EV-mediated intercellular communication: adipose tissue releases pathological EVs that propagate insulin resistance, hepatic steatosis, vascular dysfunction, and systemic inflammation. This insight opens a therapeutic avenue: if disease is driven by aberrant EV signaling, then restoring healthy EV crosstalk or delivering engineered EVs carrying corrective molecular cargo represents a mechanistically grounded, precision-medicine approach to obesity and its complications. This section synthesizes the rationale, engineering strategies, cell sources, therapeutic applications, manufacturing challenges, regulatory landscape, and clinical trial status of EV-based therapeutics, providing a comprehensive translational roadmap.

### 7.1. Why Engineer EVs? The Therapeutic Rationale

Natural EVs from healthy donors already demonstrate therapeutic efficacy: lean adipocyte EVs improve glucose tolerance and insulin sensitivity when administered to obese mice, and MSC-derived EVs exert anti-inflammatory, anti-fibrotic, and pro-regenerative effects across multiple metabolic targets [17,82]. However, natural EVs face critical limitations: insufficient targeting specificity, rapid hepatosplenic clearance, limited cargo potency, and batch-to-batch variability in yield and composition. Engineered EVs overcome these constraints by combining the intrinsic biological advantages of EVs, low immunogenicity, biodegradability, absence of genomic integration risk, and ability to traverse biological barriers, with precision-engineered surface modifications and therapeutic cargo [83,84]. Unlike viral vectors, EVs do not integrate into the host genome and elicit minimal adaptive immune responses, making them a safer platform for repeated therapeutic administration.

### 7.2. Engineering Strategies

EV engineering broadly encompasses three non-exclusive strategies: donor cell engineering, post-isolation surface modification, and active cargo loading.

Donor cell engineering involves transfecting or transducing parent cells (typically MSCs or macrophages) with plasmids or mRNA constructs encoding therapeutic miRNAs or proteins prior to EV isolation. Overexpression of anti-inflammatory miR-223 and miR-146a in donor MSCs results in secreted EVs that carry higher concentrations of these regulatory molecules, thereby enhancing immunomodulatory potency. Similarly, donor cells can be engineered to overexpress adiponectin, FGF21, or AMPK-activating constructs, embedding these metabolic regulators within the EV cargo [85]. A compelling proof-of-concept is provided by engineered EVs carrying surface FGF21 alongside encapsulated miR-223, which have demonstrated combinatorial efficacy in reducing hepatic steatosis, inflammation, and fibrosis in MASH models, outperforming single-agent approaches [85].

Surface modification strategies aim to redirect EV biodistribution toward target tissues. Peptide conjugation—such as attachment of adipose-homing peptides (e.g., CP05) to the EV surface, enables selective accumulation in visceral fat depots. Bone morphogenetic protein 7 (BMP7) delivery to white adipose tissue via surface-engineered EVs induces WAT browning and thermogenic reprogramming, representing a novel non-pharmacological approach to adiposity reduction [86]. PEGylation of EV surfaces extends circulation half-life by reducing opsonization and phagocytic clearance, while antibody or aptamer conjugation confers organ-specific targeting—liver-targeting EVs for MASH, adipose-targeting EVs for obesity, and pancreas-targeting EVs for beta-cell preservation [87].

Cargo loading methods include sonication, electroporation, and passive incubation. Electroporation is most commonly employed for nucleic acid loading (miRNA mimics, siRNA, mRNA) as it transiently permeabilizes the EV membrane. Passive loading exploits concentration gradients for small hydrophobic molecules. Synthetic EV mimetics—hybrids of liposomal membranes and EV membrane components—offer improved scalability and GMP compliance while retaining some biological targeting properties of natural EVs [83].

### 7.3. Therapeutic Cell Sources

The choice of EV donor cell determines the baseline therapeutic phenotype and tissue-homing behavior of the resulting EVs.

Mesenchymal stem cell-derived EVs (MSC-EVs) represent the most extensively studied platform. MSC-EVs are naturally anti-inflammatory, carry miRNAs and proteins that suppress NF-κB signaling, promote M2 macrophage polarization, and demonstrate efficacy in improving glucose tolerance, reducing hepatic steatosis, and activating the AMPK–SIRT1–PGC-1α axis in recipient metabolic tissues [82,88]. An umbrella review of preclinical MSC-EV studies confirmed consistently high efficacy across liver, metabolic, renal, and cardiovascular disease categories, with MSC-EVs reducing inflammation and apoptosis while promoting tissue regeneration [89].

Human Wharton’s jelly MSC-derived EVs (hWJMSC-EVs) from umbilical cord stroma represent a particularly attractive source due to their high proliferative capacity, superior immunosuppressive properties, absence of ethical concerns, and lack of donor obesity-related functional impairment. hWJMSC-EVs have demonstrated therapeutic efficacy in a metabolic syndrome rat model, significantly reducing fasting blood glucose, triglycerides, and blood pressure while improving lung and renal histopathology—effects that were dose-dependent [90]. Their cargo naturally includes bioactive molecules relevant to the FGF21–adiponectin axis, making them mechanistically suited for obesity-related MASH.

Adipose-derived stem cell EVs (ADSC-EVs) exhibit inherent adipose tissue-homing properties and have been shown to attenuate adipose inflammation, promote M2 macrophage polarization via STAT3-ARG1 signaling, induce WAT browning, and improve metabolic homeostasis in obese mice [91]. However, ADSC-EVs from obese donors exhibit impaired angiogenic and therapeutic potential due to reduced exosomal miR-126 content, underscoring the critical importance of donor metabolic status [92]. Lean-donor ADSC-EVs or those from young donors are functionally superior and represent the preferred source for therapeutic applications.

Adipose progenitor cell-derived EVs (APC-EVs) from young donors effectively suppress M1 adipose tissue macrophage polarization via miR-145-5p transfer, thereby preventing obesity-associated adipose inflammation. The miR-145-5p delivered by these EVs inhibits L-selectin expression in macrophages, blocking NF-κB-driven M1 polarization [80]. Targeted liposomes delivering miR-145-5p mimics effectively prevented midlife obesity in a mouse model, demonstrating the translational potential of this mechanism.

Regulatory T cell (Treg)-derived EVs are inherently immunosuppressive and may offer a cellular approach to restraining adipose tissue inflammation without the nonspecific systemic immunosuppression associated with pharmacological agents. M2 macrophage-derived EVs from lean donors carry anti-inflammatory cargo that restores insulin sensitivity and endothelial function, representing an intercellular therapy that repositions pathological macrophage-driven EV signaling in obesity [11].

### 7.4. Therapeutic Applications: Preclinical and Clinical Evidence

The following evidence is derived primarily from preclinical cell-based and animal model studies; findings from early-phase human clinical trials are discussed separately in Section 7.8. In glucose intolerance and T2DM, MSC-EVs have been shown to restore IRS-1 and protein kinase B phosphorylation, promote GLUT4 translocation in skeletal muscle, preserve beta-cell insulin secretion, and maintain glucose homeostasis by enhancing glycogen storage in obese rodent models [88]. The AMPK–SIRT1–PGC-1α pathway is a key effector of these metabolic improvements, mirroring the actions of insulin-sensitizing agents but without their adverse effects. In MASH, EV-based therapies targeting hepatic inflammation and fibrosis have demonstrated remarkable preclinical efficacy: hWJMSC-EVs reduce steatosis scores, suppress HSC activation, diminish collagen deposition, and improve gut barrier function, reducing endotoxemia-driven portal inflammation [82,90]. ADSC-EVs further suppress adipose macrophage M1 programs, promote healthy adipocyte hyperplasia, and restore M2 immune balance, leading to improved systemic insulin sensitivity in obese murine models [91]. In the vascular compartment, lean macrophage-derived EVs restore endothelial fibrinolytic function and attenuate prothrombotic signaling, suggesting a role in preventing obesity-associated atherothrombotic events, though these findings await confirmation in human studies [70].

### 7.5. Biodistribution and Targeting Challenges

Unmodified systemically administered EVs undergo rapid hepatosplenic accumulation via scavenger receptor-mediated phagocytosis, limiting tissue-specific delivery and reducing the fraction that reaches adipose tissue or the pancreas. PEGylation extends circulation time by shielding EVs from opsonins, while peptide, antibody, and aptamer ligands redirect EVs toward target organs [87,92]. Organ-specific targeting via RNA cargo, in which the RNA payload guides EVs to cells with complementary receptors, represents an emerging targeting paradigm. For MASH therapy, liver-targeting EVs functionalized with hepatocyte-binding ligands (e.g., GalNAc derivatives targeting ASGR1) represent a logical development given the established role of ASGR1 in hepatocyte uptake.

### 7.6. Manufacturing: The Critical Bottleneck

The clinical translation of EV therapeutics faces a defining manufacturing challenge that has no parallel in conventional drug development. The field currently lacks standardized isolation protocols, and different methods yield EV preparations with dramatically different compositions and purities. Ultracentrifugation achieves the highest purity but causes mechanical damage to EV membranes; size-exclusion chromatography better preserves EV function but yields variable purity; precipitant-based methods sacrifice purity for speed and scalability [93,94]. The consequent inconsistency in particle-to-protein ratios (typically ranging from 3 × 10^8^ to 1 × 10^10^ particles/μg protein across preparations) directly compromises batch-to-batch reproducibility, the sine qua non of pharmaceutical manufacturing [93].

Large-scale GMP-compliant EV production is achievable using hollow-fiber bioreactor systems that support high-density cell culture, with several groups having demonstrated GMP-compliant production protocols validated for clinical use [95,96]. However, current industrial-scale yields rarely exceed 10^13^ particles per liter, significantly below projected therapeutic requirements for systemic dosing—and the transition from research-grade to GMP-grade reagents can alter EV molecular composition, complicating bioactivity validation [96]. Quality control must encompass particle size distribution by nanoparticle tracking analysis (NTA), protein content by BCA assay, cargo quantification by RT-qPCR, functional bioactivity assays, endotoxin testing, and sterility verification.

Storage and shipping represent additional practical constraints. Lyophilization (freeze-drying) has emerged as a viable strategy for extending shelf life while preserving EV biological activity, enabling global distribution without continuous cold-chain infrastructure. The overall manufacturing cost currently ranges from $5000 to $50,000 per therapeutic dose, well beyond the feasibility threshold for chronic metabolic disease management. Achieving the target cost of $500–$2000 per dose will require a combination of bioreactor optimization, process intensification, and potentially the substitution of natural EVs with synthetic mimetics or plant-derived nanoparticles for standardizable applications [83,95].

### 7.7. Regulatory Pathways and Evolving Guidance

The FDA classifies engineered EVs as biologics, subjecting them to complex chemistry, manufacturing, and controls (CMC) requirements similar to those for cell and gene therapy products. Comprehensive toxicology packages, including genotoxicity, immunogenicity, and repeat-dose toxicity studies in relevant animal models, are required prior to first-in-human trials. Preclinical biodistribution studies must characterize EV clearance kinetics, tissue distribution, and persistence. The evolving regulatory guidance from the International Society for Extracellular Vesicles (ISEV), codified in the MISEV2023 guidelines, provides a scientific framework for EV characterization standards, but regulatory agency-specific technical guidelines for EV-based therapeutics are still under development in both the US and the EU [97]. In Europe, EVs are generally not classified as Advanced Therapy Medicinal Products (ATMPs) unless they incorporate gene therapy components, creating jurisdictional variability that complicates multinational clinical development programs [97]. The “hospital exemption” pathway in certain EU jurisdictions permits non-commercial clinical use of EV therapeutics under defined conditions, potentially facilitating earlier proof-of-concept human studies.

### 7.8. Clinical Trial Landscape

First-in-human EV clinical trials have demonstrated a favorable safety profile. The STEM-OM trial in China evaluated allogeneic MSC-EVs for the management of osteoarthritis and reported no serious adverse events. The HOPE trial in the UK assessed MSC-EVs for acute respiratory distress syndrome (ARDS), confirming safety with modest efficacy signals [84]. Clinical trials evaluating MSC-EVs in T2DM are ongoing at multiple sites, and trials examining adipose progenitor cell-derived EVs for metabolic obesity are in preparation. A systematic review of EV-related clinical trials confirmed that MSC-derived EVs are the most commonly explored therapeutic platform, with safety well established and efficacy data emerging across diverse conditions [84]. Engineered EVs carrying specific therapeutic payloads—such as FGF21/miR-223 combinations for MASH, or AMPK-activating cargoes for T2DM—are approaching Phase I evaluation, representing the next generation of obesity-targeted EV therapeutics.

### 7.9. Combination Strategies: Synergism with Standard Therapies

The therapeutic landscape is increasingly recognizing that EVs may be most powerful not as monotherapies but as components of multimodal strategies. EVs combined with GLP-1 receptor agonists produce synergistic improvements in weight loss and insulin sensitivity beyond either agent alone, potentially enabling dose reduction of pharmacological agents. EVs combined with time-restricted feeding enhance adipose tissue remodeling by amplifying the metabolic signaling elicited by circadian nutrient timing. EVs combined with cold exposure potentiate BAT activation and thermogenic browning of WAT, and EVs combined with structured exercise programs enhance skeletal muscle mitochondrial biogenesis by amplifying exercise-induced myokine signaling [86]. These combinatorial approaches leverage the multitarget nature of EV cargo to address the multifactorial pathophysiology of obesity.

### 7.10. Personalized EV Medicine: Precision Therapeutics for Obesity

The convergence of EV profiling technologies, multi-omics, and artificial intelligence opens the path toward personalized EV medicine. Pre-treatment EV signatures, encompassing miRNA profiles, subpopulation ratios, and cargo proteomics, can identify molecular responders to specific EV-based or pharmacological interventions, enabling patient stratification before committing to a therapeutic course. Payload selection can be tailored based on the patient’s individual EV profile: a patient with dominant hepatic steatosis signaling (high EV miR-122) may benefit from liver-targeted FGF21-EVs, whereas one with predominant adipose inflammation (high M1-EV/M2-EV ratio) may be better served by M2 macrophage-derived EVs or APC-EVs carrying miR-145-5p. Sex-stratified EV formulations that account for the hormonal modulation of adipose EV biology may further optimize therapeutic responses across sexes. Finally, tissue-specific engineering—directing EVs to the liver in MASH, to visceral adipose tissue in central obesity, or to pancreatic islets in T2DM, will maximize on-target efficacy while minimizing off-target effects [80,86,92].

### 7.11. Remaining Challenges and Future Directions

Despite remarkable progress, several key challenges must be resolved before EV therapeutics achieve mainstream clinical adoption. Immune clearance of allogeneic EVs, even with low immunogenicity, poses a barrier to repeated dosing; solutions being explored include hypoimmunogenic “universal donor” cells and autologous EV manufacturing from the patient’s own cells. Batch variability remains a manufacturing Achilles’ heel; AI-assisted quality prediction models are being developed to identify out-of-specification batches before product release. Long-term therapeutic durability is unknown for most EV formulations; repeated dosing schedules and combination therapies may address waning efficacy. Cost remains the dominant commercial barrier; synthetic mimetics, plant-derived nanoparticles, and point-of-care EV production platforms represent potential avenues for cost reduction that could bring EV therapeutics to scale [93,96].

Emerging evidence from 2024–2025 also highlights plant-derived nanoparticles and bacterial outer-membrane vesicles as potentially cost-effective, scalable alternatives to mammalian cell-derived EVs, with early studies demonstrating anti-inflammatory and metabolic benefits in preclinical obesity models.

Several important translational limitations must also be acknowledged. First, substantial interspecies differences exist in EV cargo composition, clearance kinetics, and receptor expression between rodent models and humans, meaning that preclinical findings may not directly translate to clinical efficacy. Second, considerable methodological heterogeneity in EV isolation, ranging from ultracentrifugation to size-exclusion chromatography and precipitation kits, across published human studies, limits cross-study comparability and biomarker reproducibility. Third, while early-phase clinical data are promising, the field currently lacks large, well-powered, randomized controlled trials in human obesity populations. These gaps must be resolved before EVs can be credibly positioned as tools for precision obesity medicine in routine clinical practice.

## 8. Conclusions

Extracellular vesicles have emerged as fundamental mediators of intercellular and interorgan communication, and the evidence reviewed in this article supports the view that obesity involves, at its molecular core, a profound dysregulation of this communication. The pathological EVs released by hypertrophied adipocytes, inflamed macrophages, stressed hepatocytes, and dysfunctional endothelial cells propagate metabolic disease across organ systems—converting a local adipose tissue insult into systemic insulin resistance, hepatic steatohepatitis, vascular dysfunction, and cardiovascular risk. This EV-mediated disease propagation is not merely a correlate of obesity but an active mechanistic driver, as demonstrated by the ability of obese-derived EVs to induce full metabolic disease phenotypes in lean recipients, and the capacity of lean-derived EVs to partially reverse these same phenotypes.

The recognition of EVs as pathophysiological mediators opens two complementary translational frontiers. First, the tissue-specific, cargo-rich, and dynamically responsive nature of circulating EVs makes them a uniquely powerful biomarker platform—a window into the functional state of adipose tissue, liver, vasculature, and immune compartments that cannot be matched by traditional serum analytes. EV miRNA signatures (particularly miR-122/192 for hepatic disease, miR-27a/b for insulin resistance, and miR-155 for adipose macrophage activation), subpopulation ratios (PMV/EMP for thrombotic and endothelial risk), and TF+ EV levels for cardiovascular prognosis collectively constitute the foundation of an “obesity EV biomarker panel” that could transform how obesity severity, metabolic risk, and therapeutic response are assessed in clinical practice. The integration of machine learning into multi-parameter EV data will accelerate this diagnostic revolution.

Second, the very same EV biology that drives disease provides the blueprint for its correction. MSC-derived EVs, hWJMSC-EVs, ADSC-EVs, and APC-EVs each carry natural therapeutic cargo that partially restores the metabolic communication disrupted by obesity. Engineering these natural platforms—overloading them with anti-inflammatory miRNAs, metabolic regulators (FGF21, AMPK activators, adiponectin), or tissue-targeting surface ligands amplifies their potency and directs it precisely where needed. The combination of EV therapeutics with GLP-1 agonists, exercise, dietary interventions, and metabolic surgery represents a new paradigm: multi-modal, mechanism-based treatment of obesity that moves beyond symptom management toward restoration of healthy intercellular signaling.

Manufacturing and regulatory maturation are proceeding in parallel with scientific discovery. GMP-compliant EV production using hollow-fiber bioreactor systems has been demonstrated; Phase I clinical trials of MSC-EVs confirm a robust safety profile; and ISEV MISEV2023 guidelines provide an evolving quality framework. The primary remaining barriers, cost, batch variability, and targeted biodistribution, are being actively addressed through synthetic mimetics, AI-assisted quality prediction, and surface engineering.

In synthesis, extracellular vesicles bridge the molecular mechanisms of obesity from the cellular to the systemic level. By understanding their pathophysiological roles, we gain mechanistic insight into how obesity spreads its metabolic damage across the body. By harnessing their therapeutic potential, we acquire a new class of precision biologics capable of interrupting this cascade at its source. The translation of EV biology from the bench to the clinic in metabolic disease is no longer a distant aspiration; it is an advancing reality. The goal of restoring healthy EV crosstalk in individuals with obesity, and thereby achieving genuine disease remission rather than symptomatic control, defines one of the most compelling frontiers in twenty-first century precision medicine.

## Figures and Tables

**Table 1:** Key extracellular vesicle cargo alterations in obesity and their functional effects.

Cargo Type	Molecule	Cellular Source	Functional Effect in Obesity	Key Refs.
miRNA	miR-34a	Adipocytes	Inhibits M2 macrophage polarization; amplifies adipose tissue inflammation	[10]
	miR-155	ATMs (M1)	Suppresses PPARγ; impairs insulin signaling in liver and muscle	[11]
	miR-29a	ATMs (M1)	Downregulates insulin receptor substrate; promotes insulin resistance	[11]
	miR-122, miR-192	Adipocytes	Modulate hepatic lipogenesis and gluconeogenesis in distant organs	[12]
	miR-27a	Adipocytes	Represses PPARγ in skeletal muscle; induces peripheral insulin resistance	[24]
Protein	ER stress markers	Adipocytes	Elevated in obese EVs; reflect adipocyte metabolic stress; may propagate UPR in recipient cells	[15]
	Mitochondrial components	Stressed adipocytes	Exported via EVs as a stress-relief mechanism; may impair cardiomyocyte function	[15]
Lipid	Ceramides	Adipocytes	Activate pro-inflammatory TLR4 signaling in macrophages	[9]
	Phosphatidylserine (PS)	Multiple cell types	Externalized on the EV surface; provides a catalytic platform for coagulation factor assembly	[26]
	Cholesterol	Adipocytes	Altered cholesterol efflux capacity of circulating EVs in obesity	[27]
Other	Tissue factor (TF)	Platelets, monocytes, and endothelium	Initiates the extrinsic coagulation cascade; elevated in obesity; drives a prothrombotic state	[27]

Abbreviations: ATMs, adipose tissue macrophages; ER, endoplasmic reticulum; PPARγ, peroxisome proliferator-activated receptor gamma; TLR4, Toll-like receptor 4; TF, tissue factor; UPR, unfolded protein response.

**Table 2:** EV subpopulations in obesity: cellular origin, pathological roles, and therapeutic opportunities.

EV Subpopulation	Size Range	Key Cargo	Pathological Role in Obesity	Therapeutic Opportunity
Adipocyte-derived EVs (AEVs)	30–1000 nm	miR-34a, ceramides, mitochondrial fragments, ER stress proteins	Promote adipose inflammation (M2 suppression); transfer metabolic stress to the heart and pancreas; VAT > SAT inflammatory profile	Biomarkers of adipose dysfunction; targeting VAT-specific AEVs to reduce systemic inflammation [8,13,14,17]
M1 macrophage EVs	30–150 nm	miR-155, miR-29a	Transfer insulin resistance to the liver, muscle, and adipocytes; amplify pro-inflammatory ATM polarization	Selective depletion to interrupt insulin resistance propagation; blocking miR-155 delivery [8,9,23]
M2 macrophage/ADSC EVs	30–150 nm	miR-690, anti-inflammatory cytokines	Protective: improve insulin sensitivity; promote M2 polarization and WAT browning	Engineered delivery vehicles for anti-inflammatory and insulin-sensitizing miRNAs [9,20]
Platelet-derived MVs (PMVs)	100–1000 nm	Tissue factor, P-selectin, pro-adhesion molecules	Activate endothelium; promote pro-adhesive, pro-thrombotic vascular phenotype; elevated proportionally with BMI	PMV depletion or neutralization to reduce thrombotic risk in high-risk obese patients [16]
Endothelial MPs (EMPs)	100–1000 nm	Endothelin-1, MIF, phosphatidylserine	Mark and amplify endothelial damage; paracrine feed-forward vascular injury; coagulation scaffold	Circulating EMP levels as early biomarkers of cardiovascular risk in obesity [5,17]
TF-positive EVs	Variable	Tissue factor (TF), phosphatidylserine	Initiate extrinsic coagulation; key mechanistic link between obesity and VTE/arterial thrombosis	Anti-TF antibody-conjugated capture to selectively remove procoagulant EVs [16]
Apoptotic bodies	>1000 nm	DAMPs, bioactive lipids, nuclear fragments	Released from dying adipocytes, activate macrophage PRRs; perpetuate crown-like structure inflammation	Dual role: pathogenic DAMPs vs. regenerative potential (restore macrophage homeostasis in liver) [2,25]

Abbreviations: ADSC, adipose-derived stem cell; AEVs, adipocyte-derived extracellular vesicles; BMI, body mass index; DAMPs, damage-associated molecular patterns; EMPs, endothelial microparticles; MIF, macrophage migration inhibitory factor; PMVs, platelet-derived microvesicles; PRRs, pattern recognition receptors; SAT, subcutaneous adipose tissue; TF, tissue factor; VAT, visceral adipose tissue; VTE, venous thromboembolism; WAT, white adipose tissue.

## Data Availability

No new data were created or analyzed in this study. Data sharing is not applicable to this article.
